# Synthesis and characterization of mesoporous zinc oxide nanoparticles and evaluation of their biocompatibility in L929 fibroblasts

**DOI:** 10.1002/cre2.844

**Published:** 2024-01-31

**Authors:** Zahra Jowkar, Ali Moaddeli, Fereshteh Shafiei, Tara Tadayon, Seyed Ahmadreza Hamidi

**Affiliations:** ^1^ Oral and Dental Disease Research Center, Department of Operative Dentistry, School of Dentistry Shiraz University of Medical Sciences Shiraz Iran; ^2^ Legal Medicine Research Center Legal Medicine Organization Tehran Iran; ^3^ Department of Operative Dentistry, School of Dentistry Shiraz University of Medical Sciences Shiraz Iran

**Keywords:** biocompatibility, mesoporous, zinc oxide nanoparticles

## Abstract

**Objectives:**

This study aimed to synthesize and characterize mesoporous zinc oxide nanoparticles (ZnO NPs) and also to evaluate the cytotoxicity of mesoporous ZnO NPs on L929 mouse fibroblast cell lines using 3‐(4,5‐ dimethylthiazol‐2‐yl)‐2,5‐diphenyl tetrazolium bromide (MTT) assay.

**Materials and Methods:**

The synthesized mesoporous ZnO NPs were extensively characterized using X‐ray diffraction analysis (XRD), transmission electron microscopy (TEM), Brunauer–Emmett–Teller (BET) analysis, field emission scanning electron microscopy (FESEM), energy dispersive X‐ray spectra (EDAX), Fourier‐transform infrared spectroscopy (FTIR), and dynamic light scattering (DLS). The cytotoxicity of mesoporous ZnO NPs was assessed by MTT assay. The study groups for cytotoxicity assay were normal saline, 0.1% calcined mesoporous ZnO NP solution, 1% calcined mesoporous ZnO NP solution, 0.1% noncalcined mesoporous ZnO NP solution, 1% noncalcined mesoporous ZnO NP solution, 0.1% ZnO NP solution, 1% ZnO NP solution, 2% chlorhexidine, and phosphate‐buffered saline (PBS). The percentages of mean ± standard deviation of viable cells were analyzed.

**Results:**

Characterization of mesoporous ZnO NPs revealed that all the particles were in a more or less spherical shape with a wide particle size distribution of 70–100 nm. TEM image showed the uniformed and aggregated ZnO NPs with a typical size of 10–15 nm. BET analysis showed a mesoporous structure for the prepared mesoporous ZnO NPs. According to the MTT assay, chlorhexidine had the lowest cell viability percentage. Cell viability percentages of 0.1% mesoporous ZnO NP solutions (calcined and noncalcined) were statistically, significantly higher than 0.1% ZnO NP solution (*p* < .05). Cell viability percentages of 0.1% calcined and noncalcined mesoporous ZnO NP solutions and 0.1% ZnO NP solution were statistically, significantly higher than the 1% solutions (*p* < .05).

**Conclusion:**

Mesoporous ZnO NPs exhibited less cytotoxicity against L929 mouse fibroblast cell lines compared to CHX and ZnO NPs, hence are safe to use.

## 1 INTRODUCTION

In the field of restorative dentistry, it is important to address the presence of bacteria on tooth walls after the removal of infected caries during tooth preparation. This bacterial presence poses a risk to the long‐term success of dental restorations (Kasraei et al., 2014). To mitigate the invasion and growth of bacteria and minimize the occurrence of recurrent caries, various antibacterial agents can be incorporated into the restorative materials (Arun et al., 2020; Moradpoor et al., 2021). Additionally, a suggested approach involves pretreating the prepared tooth walls with an antibacterial irrigant before placing the restoration (Ribeiro et al., 2007). Chlorhexidine (CHX) is a common cavity disinfectant in dentistry. CHX alters the function of the bacterial membrane and therefore is very effective against oral pathogenic microorganisms, including Streptococcus mutans (Kabil et al., 2017). Moreover, the inhibitory effect of CHX on the host‐derived matrix metalloproteinase (MMP) has been proposed to positively affect the bond strength longevity of adhesive restorations (Moon et al., 2010). However, the durable antibacterial efficacy of CHX is questionable because of its solubility and losing its electrostatic bonds (Ribeiro et al., 2007). Therefore, different efforts have been made to develop newer antibacterial irrigants with good biocompatibility which can be applied during dental procedures (Jowkar, Fattah, et al., 2020; Jowkar, Hamidi, et al., 2020).

Zinc oxide (ZnO) is well known for its antimicrobial properties and thus has been widely used in various dental materials, such as gutta‐percha, endodontic sealers, zinc polycarboxylate cement, and zinc oxide eugenol (Moradpoor et al., 2021; Nozari et al., 2019). Moreover, ZnO is also a chemically stable, nontoxic, and cheap material (Maltanava, 2018). The key factor for the antibacterial activity of ZnO is its affinity toward the bacterial cell. ZnO can modify bacterial cell membrane function and enzymatic activity (Moradpoor et al., 2021).

To obtain greater antibacterial activity, synthesis and application of nanoscale of ZnO particles have been investigated over the two past decades (Jowkar, Hamidi, et al., 2020; Moradpoor et al., 2021). Nanoparticles (NPs) have attracted attention in different fields of dentistry in recent years because they exhibited profound antibacterial properties with deeper penetration into dentinal tubules compared to conventional antibacterial irrigants (Shayani Rad et al., 2013). Confirmed antibacterial effects against different types of dental plaque bacteria, such as Lactobacillus and S. mutans, have been previously identified for ZnO NPs (Mirhosseini et al., 2019). ZnO NPs have been incorporated into dental restorative materials as the reinforcing and antibacterial agents to control biofilm adhesion on the surface of the dental materials (Arun et al., 2020). Additionally, ZnO NPs have been previously used for enamel and dentin pretreatments before composite bonding procedures without compromising the bond strength values (Jowkar et al. 2018). It was also demonstrated that applying ZnO NP solution as a final irrigation solution in root canal therapy enhanced the fracture resistance of the endodontically treated roots (Jowkar, Hamidi, et al., 2020). Moreover, dentin pretreatment with ZnO NPs enhanced the bond strength values of the glass ionomer cement to dentin (Jowkar, Fattah, et al., 2020).

Recently, applications of mesoporous materials have attracted attention in the field of medicine and dentistry (Yan et al., 2017; Zhang et al., 2014). A mesoporous material is a porous material with a pore size diameter between 2 and 50 nm (Asefa & Tao, 2012). Mesoporous materials possess excellent properties, such as adjustable ordered mesoporous pore size and high specific surface area (Asefa & Tao, 2012). Generally, mesoporous materials are nontoxic and demonstrate high biological compatibility (Gunduz et al., 2015). The surface or inside of the pores of mesoporous materials can be easily modified and functionalized (Gunduz et al., 2015). Moreover, their compositions, structures, and pore sizes can be optimized during synthesis (Gunduz et al., 2015). Mesoporous ZnO NPs have appropriate properties, such as high surface area, porosity volume, and crystallinity, and potential antibacterial effects making them suitable for various multifunctional therapeutic applications (Katoch et al., 2021).

Due to the drawbacks of common antibacterial irrigants in dentistry, developing newer antibacterial irrigants with good biocompatibility is of great importance. Because of the large surface area‐to‐volume ratio of nano‐sized materials, such as ZnO NPs, their antibacterial effects are much greater than the solid bulk forms (Chan et al., 2015). In this connection, mesoporous ZnO NP with much more surface area than ZnO NPs can be considered a potential antibacterial irrigant in dentistry. No previous study investigated the potential applications of mesoporous ZnO NP in dentistry. When a new dental antibacterial irrigant is introduced, its biocompatibility should be first assessed for its safe clinical application. However, to the best of the authors' knowledge, no previous study assessed the biocompatibility of mesoporous ZnO NPs using L929 mouse fibroblast cell lines. Therefore, this study aimed to evaluate the cytotoxicity of mesoporous ZnO NPs compared with ZnO NPs on L929 mouse fibroblast cell lines using 3‐(4,5‐ dimethylthiazol‐2‐yl)‐2,5‐diphenyl tetrazolium bromide (MTT) assay.

## 2 MATERIALS AND METHODS

The study protocol was approved by the Research and Ethics Committee of Shiraz University of Medical Sciences (Protocol #IR.SUMS.DENTAL.REC.1399.216). ZnO NP solutions were purchased from ASEPE Company, Tabriz, Iran. Mesoporous ZnO NP was prepared and characterized as the following.

### 2.1 Preparation of mesoporous ZnO NPs

Cetyltrimethylammonium bromide (CTMAB) was used as the template, and zinc acetate (Zn (Ac) 2 ·2H 2 O) was used as a precursor for mesoporous ZnO NPs. CTMAB and zinc acetate were purchased from Merck Company.

At first, a homogeneous solution of 1.0 g of CTMAB and 480 mL of deionized water was made at 80°C. After adding and stirring 4.92 g of Zn (Ac)2 into this solution, NaOH was used to adjust PH and alkalize the solution at 80°C for 2 h. The resulting product was filtered, washed with deionized water, and then dried at room temperature for 2 h to obtain a powder sample. These procedures resulted in the as‐synthesized noncalcined mesoporous ZnO NPs. Finally, after calcination at 500°C for 4 h, calcined mesoporous ZnO NP was obtained.

### 2.2 Characterization of mesoporous ZnO NP

All analysis procedures were conducted in Beam Gostar Taban lab, Tehran, Iran, except Fourier‐transform infrared spectroscopy (FTIR), which was conducted in the central laboratory of Shiraz University, Shiraz, Iran. The prepared mesoporous ZnO NP was stored in a plastic bag in a cool and dry place away from sunlight before analysis.

### 2.3 X‐ray diffraction analysis (XRD)

The phase structure and material identification of the mesoporous ZnO NP were studied by X‐ray diffractometer (Philips PW1730) using a nickel filtered Cu Kα radiation in the 2θ range of 10–90, at 2θ steps of 0.05, and a wavelength of 1.5406 Å. The diffraction patterns were collected between 2 h of 0.38 and 88 at a scanning rate of 0.058/min.

### 2.4 Transmission electron microscopy (TEM)

The surface morphological properties of the prepared mesoporous ZnO NPs were examined by TEM images using a Philips CM120 microscope operating at 80 kV and a linear resolution of 2.5 A.

### 2.5 Brunauer–Emmett–Teller (BET) analysis

Nitrogen adsorption/desorption isotherms of mesoporous ZnO NPs were measured by a BELSORP‐mini II adsorption porosimeter at 77.3 K after being degassed at 200°C under vacuum for 1 h. The pore size distributions were calculated according to the Barrett–Joyner–Halenda (BJH) analysis (McLaren et al., 2021). The specific surface areas were calculated using the BET method (McLaren et al., 2021).

### 2.6 Field emission scanning electron microscopy (FESEM)

The surface morphology, particle size distribution, and particle aggregation mode were assessed by FE‐SEM using a TESCAN Mira II instrument. The mesoporous ZnO NP powder was dispersed in H2O, and then the sediment was dried at room temperature before gold coating.

### 2.7 Energy dispersive X‐ray spectra (EDAX)

The composition of the elements present in the sample was confirmed by EDAX map analysis.

### 2.8 Fourier‐transform infrared spectroscopy (FTIR)

A mixture of 2 mg of mesoporous ZnO NPs with 200 mg potassium bromide (FTIR grade) was pressed into a pellet. After placing the pellet into the sample holder, FTIR spectra were recorded using Bruker Tensor 27 FT‐IR spectrometer at a resolution of 4 cm^−1^ (Malaikozhundan et al., 2017).

### 2.9 Dynamic light scattering (DLS)

The dynamic light scattering instrument (DLS, SZ100; Horiba) operating at 25°C was used to measure the hydrodynamic size and particle size distribution of the synthesized mesoporous ZnO nanoparticles. The measurements were performed in the static mode with a scattering angle of 90°.

### 2.10 Study groups for MTT assay

The study groups were as the following:

Group 1: normal saline

Group 2: 0.1% calcined mesoporous ZnO NP solution

Group 3: 1% calcined mesoporous ZnO NP solution

Group 4: 0.1% noncalcined mesoporous ZnO NP solution

Group 5: 1% noncalcined mesoporous ZnO NP solution

Group 6: 0.1% ZnO NP solution

Group 7: 1% ZnO NP solution

Group 8: 2% chlorhexidine (CHX/Consepsis, Ultradent Inc.)

Group 9: phosphate buffered saline (PBS)

### 2.11 Cytotoxicity assay

After dispersing the required amount of mesoporous ZnO NP powder in ultrapure water for each concentration (0.1% and 1%), the obtained solution was sonicated for 10 min at 20 kHz for the cytotoxicity study (Cierech et al., 2019). The cytotoxicity evaluation was performed using L929 mouse fibroblast cell lines obtained from the Iranian Biologic Resource Center.

Cell cultures were divided into nine groups with eight wells per group (*n* = 8).

Purchased cells were in Dulbecco's modified Eagle's medium (DMEM; Hi Media Labs) supplemented with 10% fetal bovine serum (FBS; Sigma), 100 g/mL of amphotericin B, 1% streptomycin/penicillin antibiotic solution (10‐mg/mL streptomycin and 10,000 unit/mL penicillin), and l‐glutamine (2 mM). The cells were incubated at 37°C in a humidified atmosphere, including 95% air and 5% CO_2_. After placing 5 × 10^3^ cells per well in a flat‐bottom 96‐well cell culture microplate, the cells were incubated for 24 h at 37°C in a humidified CO_2_ incubator.

Subsequently, 100 μL solution from each study group was added to well, and the microplate was incubated at 37°C for 24 h in a humidified atmosphere including 95% air and 5% CO_2_. The MTT assay was performed to measure cell viability (Verma et al., 2018), and the absorbance values of each well were read at 570 nm through an enzyme‐linked immunoassay (ELISA) plate reader. The percentage of viable cells in each well was calculated relative to control cells set to 100%.

### 2.12 Data analysis

The percentage of viable cells in each well was expressed as the mean ± standard deviation. To assess the data normality, the Kolmogorov–Smirnov test was performed. Since the data were normally distributed, data analysis was performed using the one‐way analysis of variance, followed by the Tukey test. All the analyses were conducted using SPSS software version 17 (SPSS Inc). *p* < .05 were considered statistically significant.

## 3 RESULTS

### 3.1 Characterization of mesoporous ZnO NPs

#### 3.1.1 FESEM and EDX‐map

**Figure 1:**
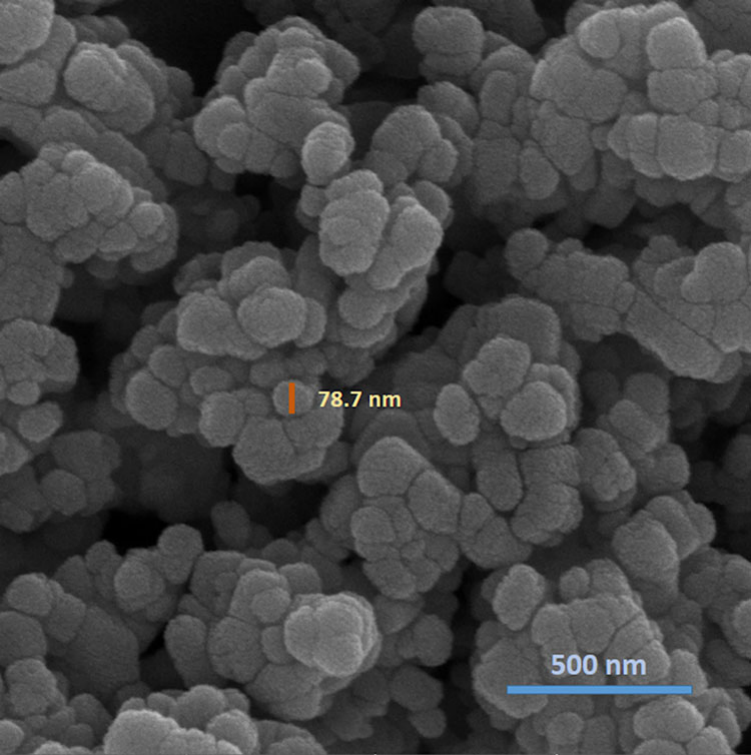
Field emission scanning electron microscopy image of mesoporous ZnO NP sample.

The morphology of the mesoporous ZnO NPs was examined via FESEM. Figure 1 shows the images at two different magnifications. It was observed that all the particles were more or less spherical with a wide particle size distribution of 70–100 nm with well‐separated grain boundaries. The particles were found to be homogeneously aggregated by very small particles, resulting in the formation of a bulky spherical structure.

**Figure 2:**
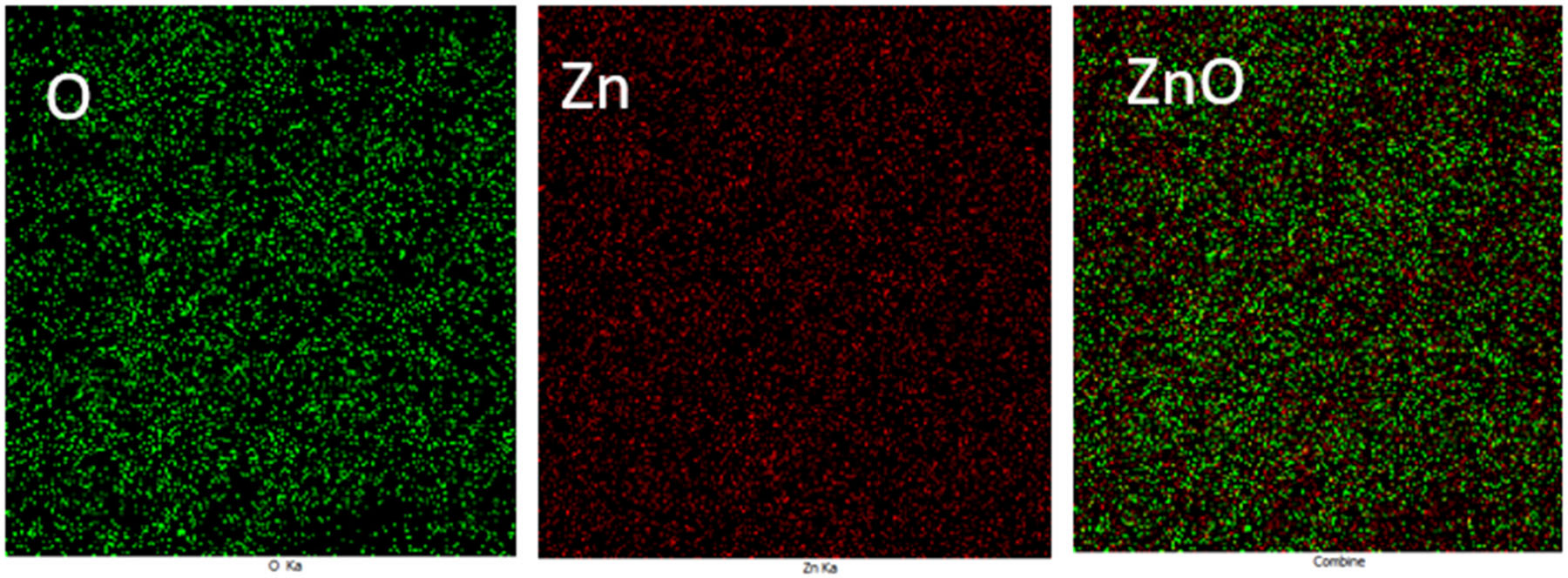
The field emission scanning electron microscopy EDX elemental mapping of Zn, O, and ZnO.

The EDX‐map analysis showed the homogeneous distribution of the Zn and O elements (Figure 2).

#### 3.1.2 TEM

**Figure 3:**
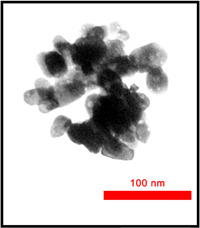
Transmission electron microscopy image of mesoporous ZnO NP sample.

Mesoporous structure of the prepared mesoporous ZnO NPs was further investigated by TEM (Figure 3). TEM image showed the uniformed and aggregated mesoporous ZnO NPs with the typical size of 10–15 nm. Many of the mesoporous ZnO NPs appeared to be quasi‐spherical. The porosity on the surface of the particles can be clearly observed. In addition, the empties or interspaces between the nanoparticles are considered as larger pores generated by the inter‐agglomeration of mesoporous ZnO NPs that confirmed the BJH analysis.

#### 3.1.3 XRD

**Figure 4:**
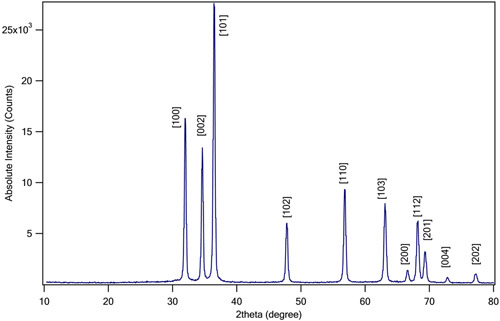
Wide‐angle XRD pattern of mesoporous ZnO NP sample.

Figure 4 displays the XRD patterns of the mesoporous ZnO nanoparticle sample. The sharp diffraction peaks illustrated in the figure at 231.992, 34.642, 36.442, 47.792, 56.842, 63.092, 66.592, 68.242, 69.342, 72.792, and 77.292° corresponding planes of [100, 002, 101, 102,110, 103, 200, 112, 201, 004] and [202], respectively, indicate high crystalline structure of the prepared mesoporous ZnO NPs. These peaks are compatible with the standard reported values with JCPDS Card no. 036‐451 (Figure 4).

**Figure 5:**
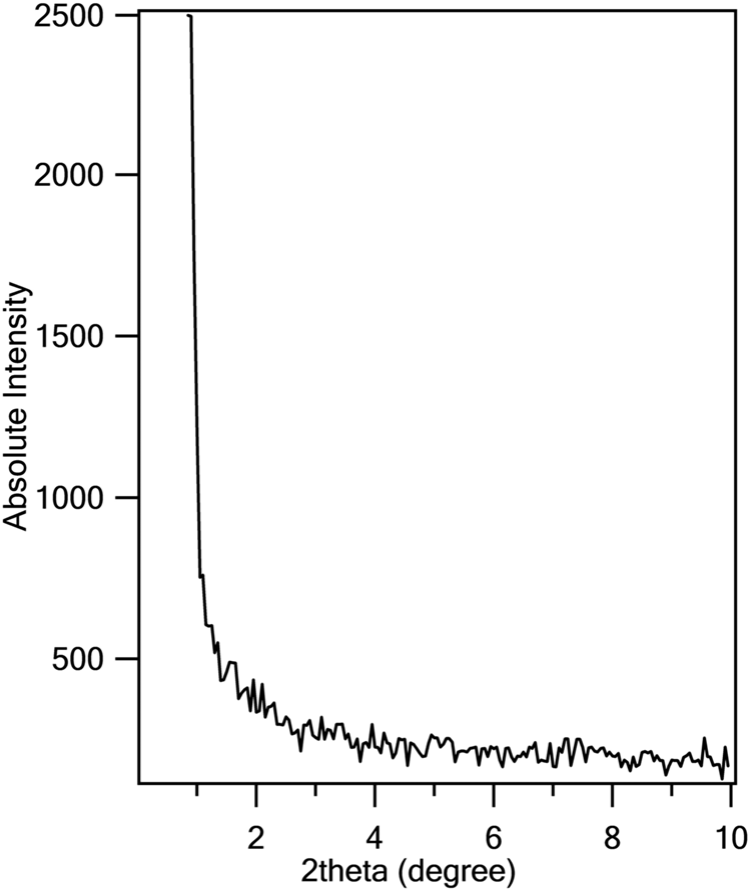
Low‐angle XRD pattern of mesoporous ZnO NP sample.

Low angle XRD pattern of mesoporous ZnO was investigated in the two theta range of 0.85–10°, and no characteristic peak was found in this range (Figure 5).

#### 3.1.4 BET isotherm and BJH

**Figure 6:**
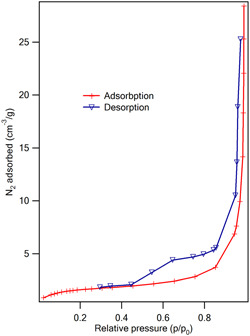
Nitrogen adsorption‐desorption isotherm of mesoporous ZnO NPs.

**Figure 7:**
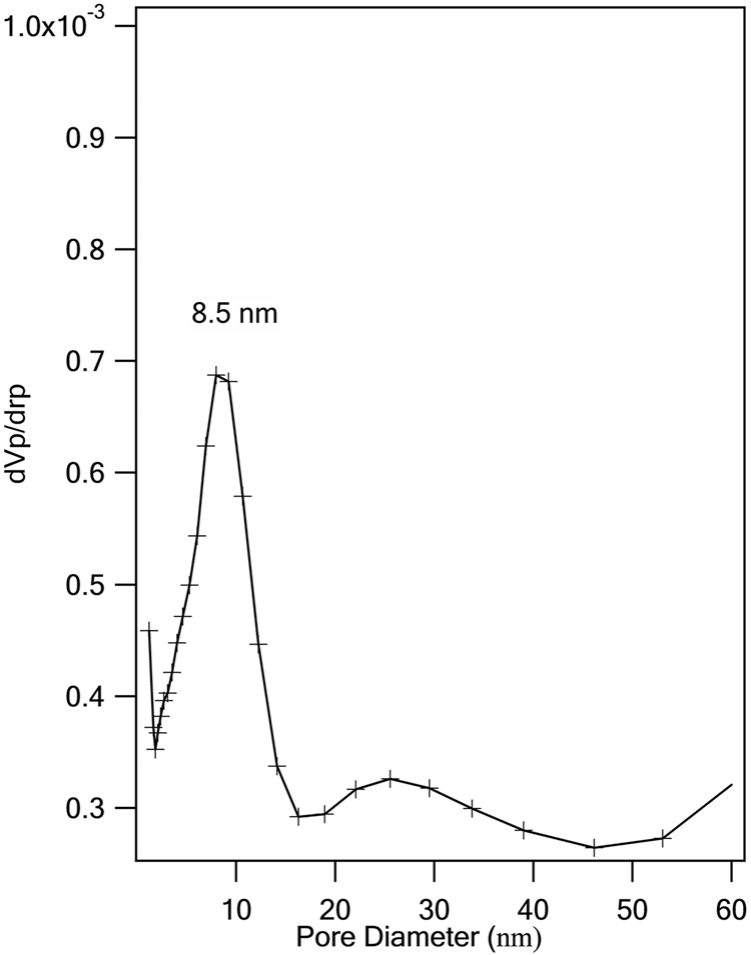
Pore size distribution plots obtained by BJH model for the adsorption/desorption branch isotherm of mesoporous ZnO NPs.

N2 adsorption/desorption analysis was performed to study the textural properties of the mesoporous ZnO NPs (Figure 6). Type IV isotherm with H3 hysteresis loop indicated that the prepared mesoporous ZnO NPs have mesoporous structures. The specific surface area calculated by the BET method was 5 m^2^/g. BJH plot was used to evaluate the pore size distribution of the sample. Figure 7 represents the BJH desorption cumulative pore size curve. The presence of two types of pores in terms of size with 8.5 and 27 nm in the mesoporous region was confirmed.

#### 3.1.5 FTIR

**Figure 8:**
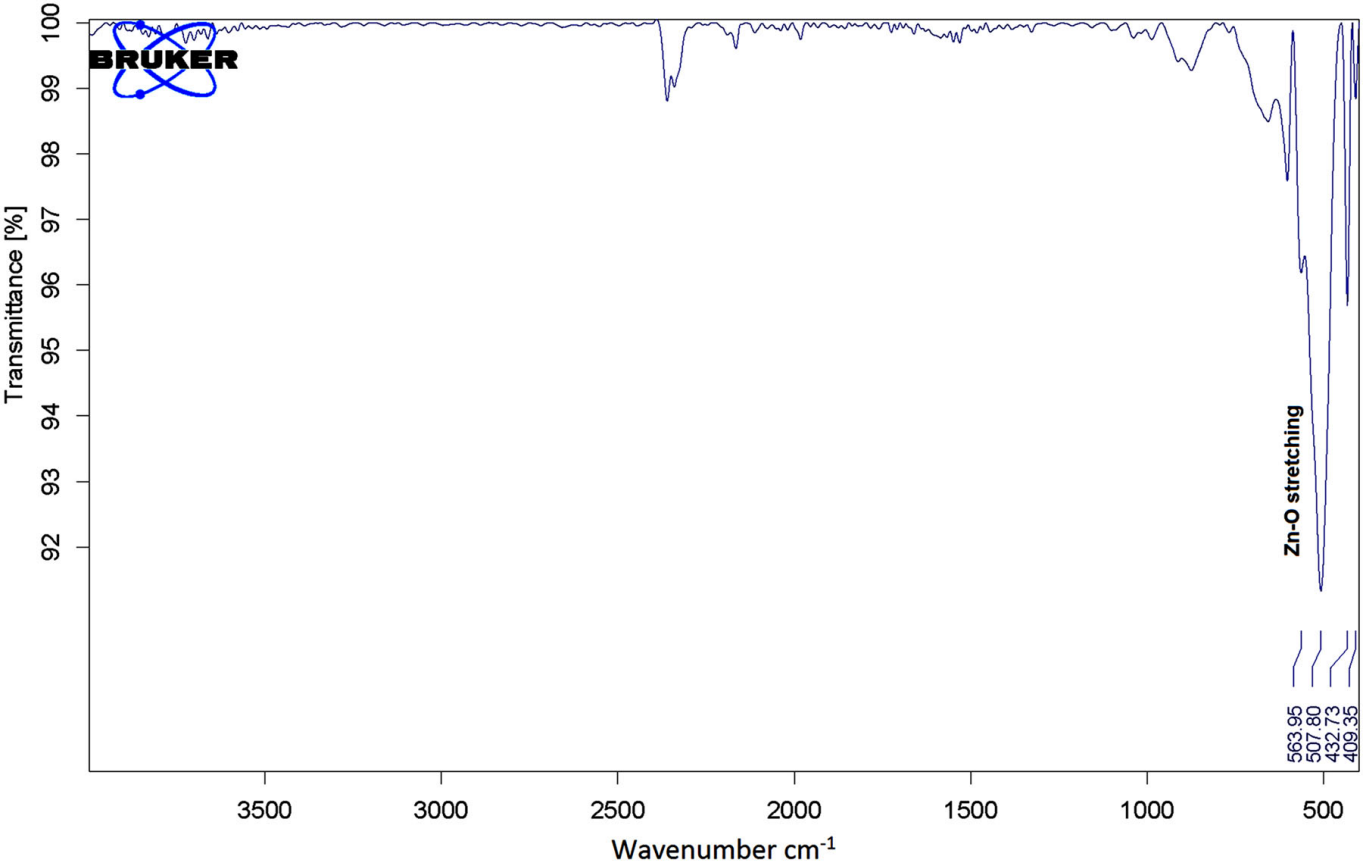
The FTIR spectra of mesoporous ZnO NPs.

The surface composition of the calcined sample was studied via FTIR, and the spectrum is shown in Figure 8. The FTIR spectra of the ZnO usually showed a characteristic absorption band between 420 and 510 cm^−1^ due to the two transverse optical stretching modes of ZnO. In the case of the prepared mesoporous ZnO nanoparticles, the peek corresponding to the stretching vibration of Zn‐O is split into two maxima, one at 508 and the second one at 433 cm^−1^, confirming the formation of ZnO.

#### 3.1.6 DLS

**Figure 9:**
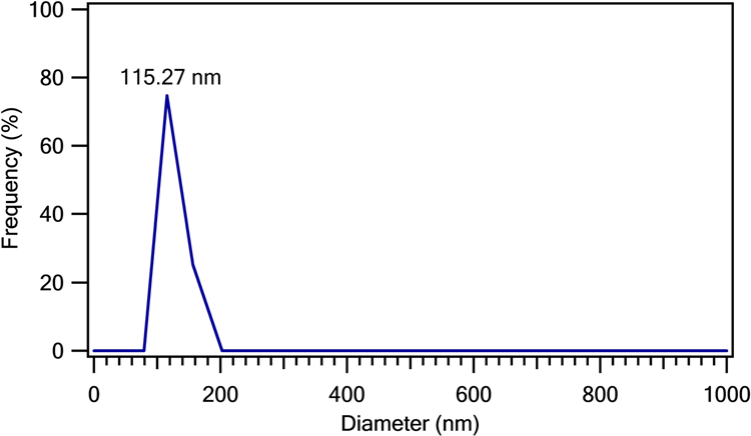
Dynamic light scattering of the mesoposrous ZnO NPs.

Size dispersion of mesoporous ZnO was measured using dynamic light scattering to confirm the average size (Figure 9). The average particle size of m‐ZnO was 115.27 nm which is confirmed by the observed particle size in SEM image.

### 3.2 MTT assay

**Figure 10:**
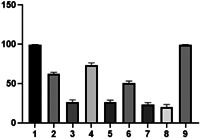
MTT viability data for L929 cells treated with different study groups. (1; group 1 (normal saline), 2; group 2 (0.1% calcined mesoporous ZnO NP solution), 3; group 3 (1% calcined mesoporous ZnO NP solution), 4; group 4 (0.1% noncalcined mesoporous ZnO NP solution), 5; group 5 (1% noncalcined mesoporous ZnO NP solution), 6; group 6 (0.1% ZnO NP solution), 7; group 7 (1% ZnO NP solution), 8; group 8 (2% CHX,) 9; group 9 (PBS)).

Table 1 displays the mean percentages of viable cells along with their standard deviations (SD). The results of the one‐way analysis of variance test indicate that there were significant statistical differences between the experimental groups (*p* = .001). To compare the groups pairwise, the Tukey test was conducted. Figure 10 presents the data regarding cell viability percentages of various study groups on L929 cells.

**Table 1:** The mean percentage of viable cells for each experimental group determined by the MTT cytotoxicity assay.

Group number	Study group	Mean cell viability % (±standard deviation)
1	Normal saline	99.31% (±0.20)
2	0.1% calcined mesoporous ZnO NP solution	62.43% (±0.75)
3	1% calcined mesoporous ZnO NP solution	26.49% (±1.48)
4	0.1% noncalcined mesoporous ZnO NP solution	73.70% (±0.96)
5	1% noncalcined mesoporous ZnO NP solution	26.28% (±1.03)
6	0.1% ZnO NP solution	50.81% (±0.93)
7	1% ZnO NP solution	23.68% (±0.73)
8	2% CHX (chlorhexidine)	20.54% (±1.07)
9	Phosphate buffered saline (PBS)	99.02% (±0.33)

According to the Tukey test, the mean cell viability of normal saline and PBS was significantly higher than the other experimental groups (*p* < .001). There was no significant difference observed between the cell viability percentages of normal saline and PBS (*p* > .05).

In comparison to all NP solutions (excluding the 1% ZnO NP solution), CHX exhibited significantly lower cell viability percentages (*p* < 0.001). However, the 1% ZnO NP solution did not demonstrate significant differences with CHX (*p* < .05).

The cell viability percentages of the 0.1% calcined and noncalcined mesoporous ZnO NP solutions, as well as the 0.1% ZnO NP solution, were significantly higher than the 1% solutions (*p* < .001). Furthermore, the cell viability percentages of the 0.1% mesoporous ZnO NP solutions were significantly higher than the 0.1% ZnO solution. However, there were no significant differences found between the cell viability percentages of the 1% mesoporous ZnO NP solutions (calcined and noncalcined), and the 1% ZnO NP solution (*p* > .05).

## 4 DISCUSSION

This study was conducted to evaluate the cytotoxicity of mesoporous ZnO NPs in comparison with ZnO NPs on L929 mouse fibroblast cell lines. According to the results of the present study, all NP solutions demonstrated higher cell viability percentages compared to CHX. The highest cell viability percentage was observed for 0.1% mesoporous ZnO NP solution.

The bacteria are not usually removed completely from the prepared tooth surfaces even after careful caries removal (Kasraei et al., 2014). Additionally, gap formation at the tooth‐restoration interface may lead to bacterial penetration and colonization at the tooth‐restoration interface, and eventually, the development of secondary caries (Khvostenko et al., 2015). Therefore, the synthesis of antibacterial materials and their applications during restorative procedures have been suggested to reduce the pathogenic impact of oral biofilms (Jowkar et al., 2018). One of the most common cavity disinfectants used in restorative dentistry is CHX (Suma, 2017). Besides its antibacterial properties, CHX demonstrates a matrix metalloproteinase (MMP)‐inhibitory effect which may positively affect bond strength durability. However, the antibacterial and MMP‐inhibitory effects might be temporary (Borges et al., 2012; Sadek et al., 2010). Moreover, CHX may have negative effects on the bond strength of the adhesive systems to tooth structure (Ercan et al., 2009). Therefore, efforts to find alternative cavity disinfectants are ongoing to overcome the disadvantages of the common previous cavity disinfectants (Jowkar, Fattah, et al., 2020).

Recently, ZnO NPs have gained significant attention in different medical and dental fields due to their exceptional antibacterial properties (Alhujaily et al., 2022; Moradpoor et al., 2021). In a previous investigation, the therapeutic potential of a nanocomposite (NC) comprising ZnO NPs and gold (Au) nanoparticles (NPs) was examined (Alhujaily et al., 2023). The NC was extensively characterized, revealing a core‐shell structure formed by the adherence of ZnO NPs onto the surface of Au NPs (Alhujaily et al., 2023). Notably, the Au/ZnO NCs exhibited improved antibacterial activity against *Escherichia coli* compared to individual Au and ZnO NPs (Alhujaily et al., 2023). Moreover, the therapeutic efficacy of the synthesized Au/ZnO NCs was assessed on human breast cancer cells and human esophageal adenocarcinoma cancer cells (Alhujaily et al., 2023). These findings indicate that the synthesized Au/ZnO NCs hold great potential for various biomedical applications (Alhujaily et al., 2023).

In a prior investigation, the green synthesis of zinc oxide nanoparticles (ZnO NPs) and their potential biomedical applications were examined (Alhujaily et al., 2022). The synthesized ZnO NPs demonstrated a wide range of medical and biotechnological uses, thanks to their favorable biocompatibility, low cytotoxicity, and cost‐effectiveness, rendering them suitable for diverse industries (Alhujaily et al., 2022). Furthermore, the study revealed that the synthesized ZnO NPs exhibited antimicrobial, anticancer, antioxidant, and anti‐inflammatory properties (Alhujaily et al., 2022). Additionally, they possessed the capacity to improve drug delivery and enable bioimaging, thereby indicating their potential for application in both medical and industrial settings (Alhujaily et al., 2022).

It has been demonstrated that the antibacterial properties of ZnO NPs are directly related to their surface area (Demir et al., 2014). Moreover, ZnO NPs demonstrated high safety and stability, good biocompatibility, low toxicity, and low cost (Moradpoor et al., 2021; Thakral et al., 2021). Therefore, ZnO NPs have drawn attention in many fields of dentistry, such as endodontics, restorative dentistry, prosthodontics, and periodontal fields (Jowkar, Hamidi, et al., 2020; Jowkar, Omidi, et al., 2020).

A remarkable decrease in biofilm formation by 85% has been previously demonstrated for teeth surfaces coated with ZnO NPs compared to the uncoated tooth (Eshed et al., 2012). A significant improvement in the antibacterial properties of resin composites containing at least 1% ZnO NPs (wt %) has been previously demonstrated (Arun et al., 2020). Moreover, bond strength, mechanical and physical properties, and antimicrobial activities of a flowable resin composite were positively affected by ZnO NP incorporation (Hojati et al., 2013). However, due to the possible negative effects of incorporation of NPs into resin composites or adhesive bonding systems, it is suggested to apply NPs as a pretreatment step during restorative procedures to benefit from their antibacterial effects (Jowkar et al., 2018, Moradpoor et al., 2021).

Recently, mesoporous materials have suggested a promising therapeutic solution to a wide variety of medical fields (Tang et al., 2012; Vallet‐Regí, 2022). Some mesoporous materials also have been previously used in dentistry (Akram et al., 2021; Chiang et al., 2014; Fan et al., 2016). Mesoporous calcium‐silicate nanoparticles loaded with CHX presented excellent anti‐E. faecalis ability, low cytotoxicity, the ability to release CHX and Ca2+/SiO3 2− ions, and in vitro remineralization (Fan et al., 2016). Therefore, mesoporous calcium‐silicate nanoparticles loaded with CHX have been applied as a new bone defect filling material for infected bone defects and as a new effective intracanal medication in dentistry (Fan et al., 2016). Moreover, mesoporous silica nanoparticles (MS NPs) were previously used to encapsulate and release CHX from resin composite (Zhang et al., 2014). Strong inhibition of *Streptococcus mutans* and *Lacticaseibacillus casei* without sacrificing materials' mechanical properties and surface integrity were observed for resin composites incorporated with MS NPs loaded with CHX (Zhang et al., 2014). Additionally, it was demonstrated that a high amount of CHX (44.62 wt. %) can be carried by pore‐expanded MS NPs (pMS NPs). Adding pMS NPs to glass ionomer cement led to significant antibiofilm potential without compromising the mechanical properties of GIC (Yan et al., 2017).

Mesoporous ZnO NPs have been previously suggested for ureteral stent fabrication because of their antibacterial effects, biodegradability, and drug elution ability (Laurenti et al., 2020). Due to their unique properties, such as high surface area, potential antibacterial effects, and high crystallinity, mesoporous ZnO NPs may be used as an antibacterial irrigant in dentistry (Laurenti et al., 2020). Besides strong antibacterial properties against cariogenic bacteria, an ideal antibacterial cavity disinfectant should have minimal cytotoxic effects on the host. Therefore, when creating a new antibacterial irrigation solution, its cytotoxicity should be first assessed due to the possible contact of the antimicrobial irrigant with tissues when used during clinical application (Generali et al., 2020). In the present study, the cell viabilities of the mesoporous ZnO NP solutions, which can be used as potential antibacterial cavity irrigants, were compared to that of CHX and normal saline.

In vitro models are initial studies that mimic the in vivo conditions and thus are commonly used to assess the cytotoxicity of a novel irrigant (ALGhanem et al., 2017). MTT assay was chosen for cytotoxicity assessment in the present study. MTT assay is a simple quantitative test designed for biocompatibility testing. MTT assay is a colorimetric method and distinguishes nonviable from viable cells by microscopic analysis. The viable active cells reduce yellow MTT to insoluble purple formazan crystal dissolved using DMSO by mitochondrial dehydrogenase enzyme. Therefore, the percentage of living cells in a solution can be quantified by this method (ALGhanem et al., 2017). Additionally, the MTT assay is a sensitive, precise, and very rapid method to measure the activity of all cell lines (Generali et al., 2020).

In the present study, normal sterile saline was used as a control group which showed a high percentage of cell viability (99.31%) after 24 h. This finding is in agreement with previous studies that consider saline as a biocompatible material (Abdeltawab et al., 2022; Nabavizadeh et al., 2018). Moreover, the cell viability percentage of normal saline was not different from PBS.

The lowest cell viability percentage was observed for CHX in the present study. Therefore, all NP solutions were more biocompatible compared to CHX, a routine cavity disinfectant in restorative dentistry.

The present study used two different concentrations of ZnO NP solutions and mesoporous ZnO NP solutions, including 1% and 0.1%. It was found that the cell viability percentages of all 0.1% NP solutions were statistically, significantly higher than that of 1% solutions. This finding was in agreement with a previous study which reported that the intensity of NPs toxicity is dependent on their concentrations (Zanette et al., 2011). Another study also showed that the toxicity of NPs is generally proportional to the amount of NPs in the test mediums, which was in line with the findings of the present study (Hashimoto et al., 2016). Moreover, an increased concentration of ZnO NPs resulted in an increased rate of cytotoxicity in a previous study (Geetha et al., 2020).

This study also compared the cell viability percentages of ZnO NP solutions and mesoporous ZnO NP solutions. According to the findings of the present study, 0.1% mesoporous ZnO NPs solutions were more biocompatible than 0.1% ZnO NPs. However, the differences in 1% concentrations were not statistically significant. Lower toxicity of the mesoporous ZnO NPs compared to that of ZnO NPs might be related to the smaller sizes of ZnO NPs compared to the mesoporous ZnO NPs. More cytotoxicity for smaller particles compared with larger ones (size effects) has also been previously reported (Hashimoto et al., 2016). According to the findings of the present study, due to the significantly lower cell viabilities of 1% NP solutions compared to those of the 0.1% NP solutions, the preferred concentration for safe clinical use seems to be 0.1% NP solutions.

Two different forms of mesoporous ZnO NP solutions, including calcined and noncalcined forms in two concentrations (1% and 0.1%), were assessed in the present study. The calcined form was obtained when mesoporous ZnO NPs were heated to remove the volatile components. The results of the present study showed no differences between the cell viability percentages of the two forms. Therefore, both of them can be used as antibacterial irrigants in dentistry. However, more studies should be performed considering their wettability, antibacterial properties, and the effect of each form on the bond strength to tooth structures to investigate the possible differences between the two forms for their clinical application.

The results of the present study showed an insight for assessing possible applications of the mesoporous ZnO NPs in different fields of dentistry such as restorative dentistry, endodontics, and periodontal therapy. According to the results of the present study, mesoporous ZnO NPs were more biocompatible compared to CHX, which is commonly used as an antibacterial irrigant in dental clinical practice. Due to the good biocompatibility of mesoporous ZnO NPs, they have the potential to be used as the antibacterial irrigant during restorative, endodontic, and periodontal treatments. Moreover, mesoporous ZnO NPs can be used as a reinforcing agent when incorporated into dental materials. However, the effects of dentin or enamel pretreatment with mesoporous ZnO NPs on the bond strength of dental materials to tooth structures and also the effect of mesoporous ZnO NP incorporation on the mechanical and physical properties of restorative or endodontic dental materials should be assessed in future studies.

This study has some limitations. First, only the cytotoxicity of the mesoporous ZnO NPs after 24 h was assessed in the present study. It would also be beneficial to study the cytotoxicity and tissue reactions for longer periods. Moreover, the cytotoxicity of mesoporous ZnO NPs should be investigated by different methods other than MTT assay. The effects of mesoporous NPs on various cell types should be investigated in future research. Moreover, more in vivo and in vitro studies assessing the antimicrobial efficacy of mesoporous ZnO NPs against different oral bacteria should be performed in the future to use these NPs in clinical practice confidently. Additionally, other properties of mesoporous ZnO NPs such as antifungal, anti‐inflammatory, and possible anticancer properties of mesoporous ZnO NPs should be investigated in future studies.

## 5 CONCLUSION

The findings of this study provide important insights into the biocompatibility of mesoporous ZnO NPs and ZnO NPs, in the context of their potential applications in dentistry. The study demonstrated that 0.1% Mesoporous ZnO NPs had lower cytotoxic effects on L929 mouse fibroblast cell lines compared to the commonly used antibacterial irrigant chlorhexidine (CHX) and 0.1% ZnO NPs, indicating their safety for use. CHX exhibited significantly lower cell viability percentages compared to all NP solutions, except for the 1% ZnO NP solution. This suggests that the 1% ZnO NP solution may possess similar cytotoxic effects as CHX. The lower concentrations of mesoporous ZnO NP solutions (0.1%) demonstrate higher cell viability percentages compared to the higher concentrations (1%). Therefore, The preferred concentration based on the biocompatibility findings is 0.1%. These results provide valuable insights into the potential application of mesoporous ZnO NPs as antimicrobial agents in various dental treatments, including endodontic, restorative dentistry, and periodontal therapies.

## AUTHOR CONTRIBUTIONS

Zahra Jowkar and Ali Moaddeli conceived the ideas; all authors collected the data; all authors analyzed the data; and Zahra Jowkar and Ali Moaddeli led the writing.

## CONFLICT OF INTEREST STATEMENT

The authors declare no conflict of interest.

## Data Availability

The data that support the findings of this study are available on request from the corresponding author.
